# *Annona muricata* (Annonaceae): A Review of Its Traditional Uses, Isolated Acetogenins and Biological Activities

**DOI:** 10.3390/ijms160715625

**Published:** 2015-07-10

**Authors:** Soheil Zorofchian Moghadamtousi, Mehran Fadaeinasab, Sonia Nikzad, Gokula Mohan, Hapipah Mohd Ali, Habsah Abdul Kadir

**Affiliations:** 1 Biomolecular Research Group, Biochemistry Program, Institute of Biological Sciences, Faculty of Science, University of Malaya, 50603 Kuala Lumpur, Malaysia; E-Mails: soheil.zorofchian@gmail.com (S.Z.M.); sonia.nikzad@gmail.com (S.N.); g.mohan@um.edu.my (G.M.); 2 Department of Chemistry, Faculty of Science, University of Malaya, 50603 Kuala Lumpur, Malaysia; E-Mails: mehranfadaei@um.edu.my (M.F.); hapipah@um.edu.my (H.M.A.)

**Keywords:** *Annona muricata*, annonaceae, acetogenins, natural products, biological activity, bioactive compounds, fruit tree

## Abstract

*Annona muricata* is a member of the Annonaceae family and is a fruit tree with a long history of traditional use. *A. muricata*, also known as soursop, graviola and guanabana, is an evergreen plant that is mostly distributed in tropical and subtropical regions of the world. The fruits of *A. muricata* are extensively used to prepare syrups, candies, beverages, ice creams and shakes. A wide array of ethnomedicinal activities is contributed to different parts of *A. muricata*, and indigenous communities in Africa and South America extensively use this plant in their folk medicine. Numerous investigations have substantiated these activities, including anticancer, anticonvulsant, anti-arthritic, antiparasitic, antimalarial, hepatoprotective and antidiabetic activities. Phytochemical studies reveal that annonaceous acetogenins are the major constituents of *A. muricata*. More than 100 annonaceous acetogenins have been isolated from leaves, barks, seeds, roots and fruits of *A. muricata*. In view of the immense studies on *A. muricata*, this review strives to unite available information regarding its phytochemistry, traditional uses and biological activities.

## 1. Introduction

Natural products, especially those derived from plants, have been used to help mankind sustain its health since the dawn of medicine. Over the past century, the phytochemicals in plants have been a pivotal pipeline for pharmaceutical discovery. The importance of the active ingredients of plants in agriculture and medicine has stimulated significant scientific interest in the biological activities of these substances [1]. Despite these studies, a restricted range of plant species has experienced detailed scientific inspection, and our knowledge is comparatively insufficient concerning their potential role in nature. Hence, the attainment of a reasonable perception of natural products necessitates comprehensive investigations on the biological activities of these plants and their key phytochemicals [2]. In a pharmaceutical landscape, plants with a long history of use in ethno medicine are a rich source of active phytoconstituents that provide medicinal or health benefits against various ailments and diseases. One such plant with extensive traditional use is *Annona muricata*. In this review, we describe the botany, distribution and ethnomedicinal uses of this plant, and we summarize the phytochemistry, biological activities and possible mechanisms of *A. muricata* bioactivities.

## 2. Botanical Description and Distribution

**Figure 1:**
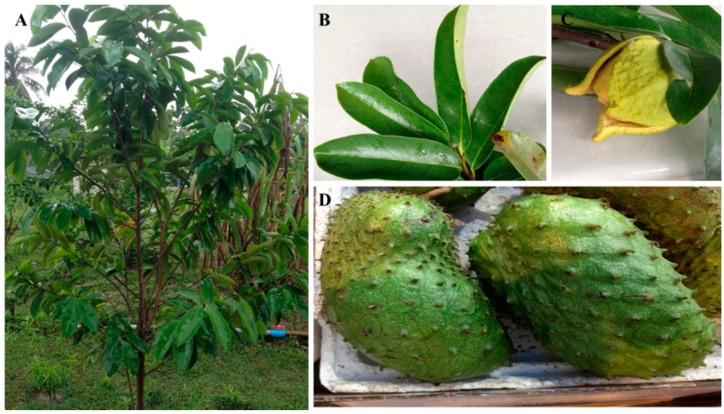
(**A**) *Annona muricata* L.; the appearance of the (**B**) leaves; (**C**) flowers and (**D**) fruits.

*A. muricata* L., commonly known as soursop, graviola, guanabana, paw-paw and sirsak, is a member of the Annonaceae family comprising approximately 130 genera and 2300 species [3,4]. *A. muricata* is native to the warmest tropical areas in South and North America and is now widely distributed throughout tropical and subtropical parts of the world, including India, Malaysia and Nigeria [5]. *A. muricata* is an evergreen, terrestrial, erect tree reaching 5–8 m in height and features an open, roundish canopy with large, glossy, dark green leaves. The edible fruits of the tree are large, heart-shaped and green in color, and the diameter varies between 15 and 20 cm (Figure 1) [6].

## 3. Ethnomedicinal Uses

All portions of the *A. muricata* tree, similar to other *Annona* species, including *A. squamosa* and *A. reticulata* are extensively used as traditional medicines against an array of human ailments and diseases, especially cancer and parasitic infections. The fruit is used as natural medicine for arthritic pain, neuralgia, arthritis, diarrhea, dysentery, fever, malaria, parasites, rheumatism, skin rushes and worms, and it is also eaten to elevate a mother’s milk after childbirth. The leaves are employed to treat cystitis, diabetes, headaches and insomnia. Moreover, internal administration of the leaf’s decoction is believed to exhibit anti-rheumatic and neuralgic effects, whereas the cooked leaves are topically used to treat abscesses and rheumatism [3,5,7]. The crushed seeds are believed to have anthelmintic activities against external and internal worms and parasites. In tropical Africa, the plant is used as an astringent, insecticide and piscicide agent and to treat coughs, pain and skin diseases. In India, the fruit and flower are employed as remedies against catarrh, while the root-bark and leaves are believed to have antiphlogistic and anthelmintic activities [8,9]. In Malaysia, the crushed leaf mixture of *A. muricata* together with *A. squamosa* and *Hibiscus rosa-sinensis* is used as a juice on the head to protect against fainting [10]. In South America and tropical Africa, including Nigeria, leaves of *A. muricata* are deployed as an ethnomedicine against tumors and cancer [8]. In addition, the anti-inflammatory, hypoglycemic, sedative, smooth muscle relaxant, hypotensive and antispasmodic effects are also attributed to the leaves, barks and roots of *A. muricata* [3,5]. In addition to ethnomedicinal uses, the fruits are widely employed for the preparation of beverages, candy, ice creams, shakes and syrups [11,12].

## 4. Phytochemistry

**Figure 2:**
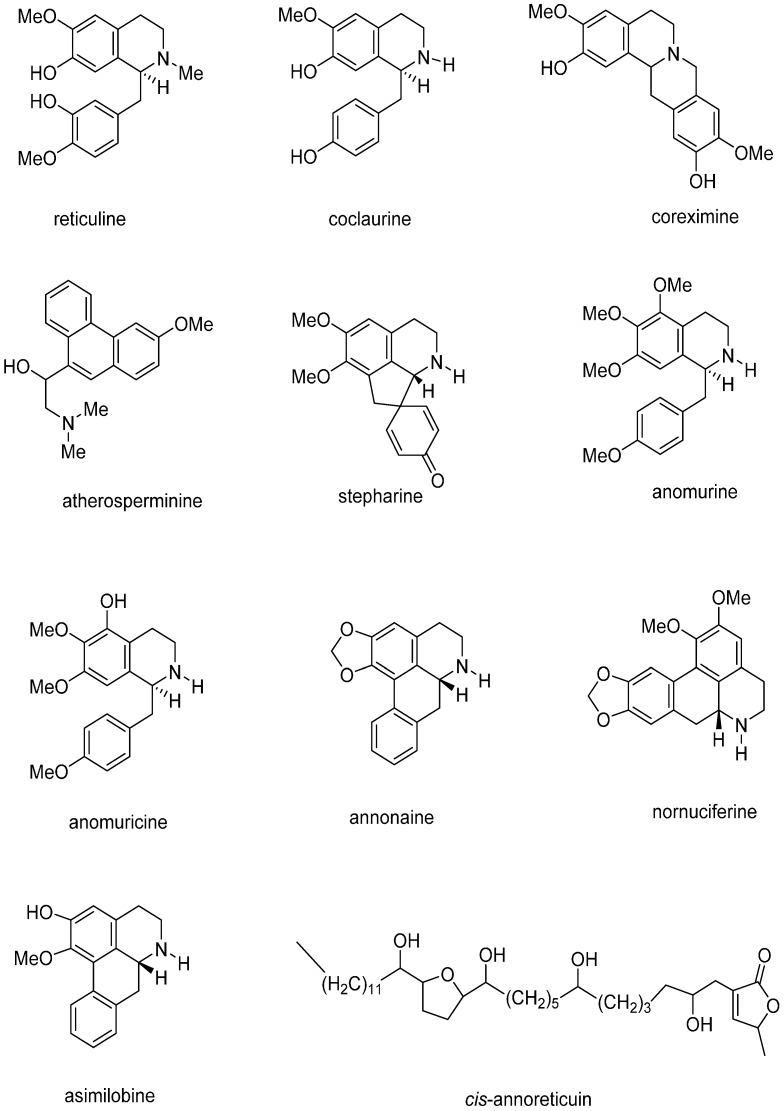

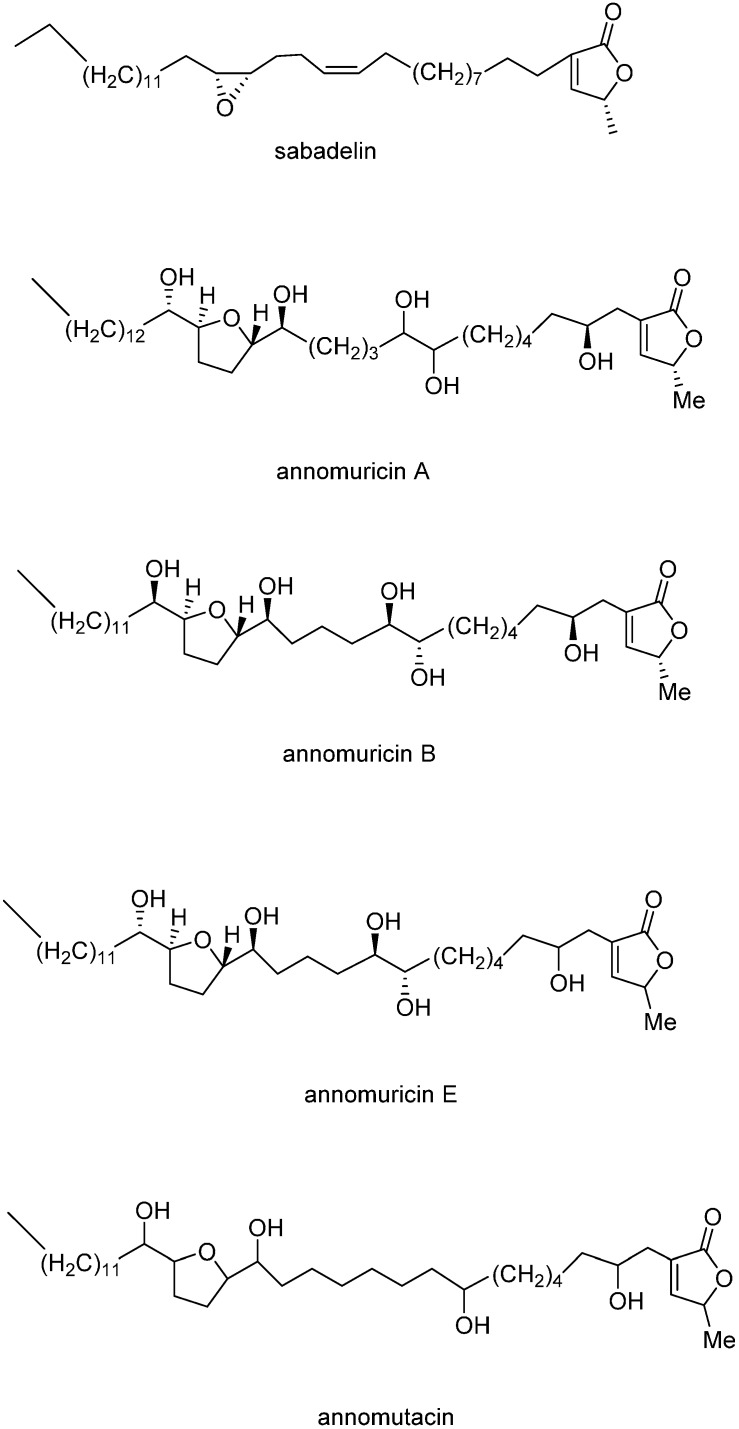

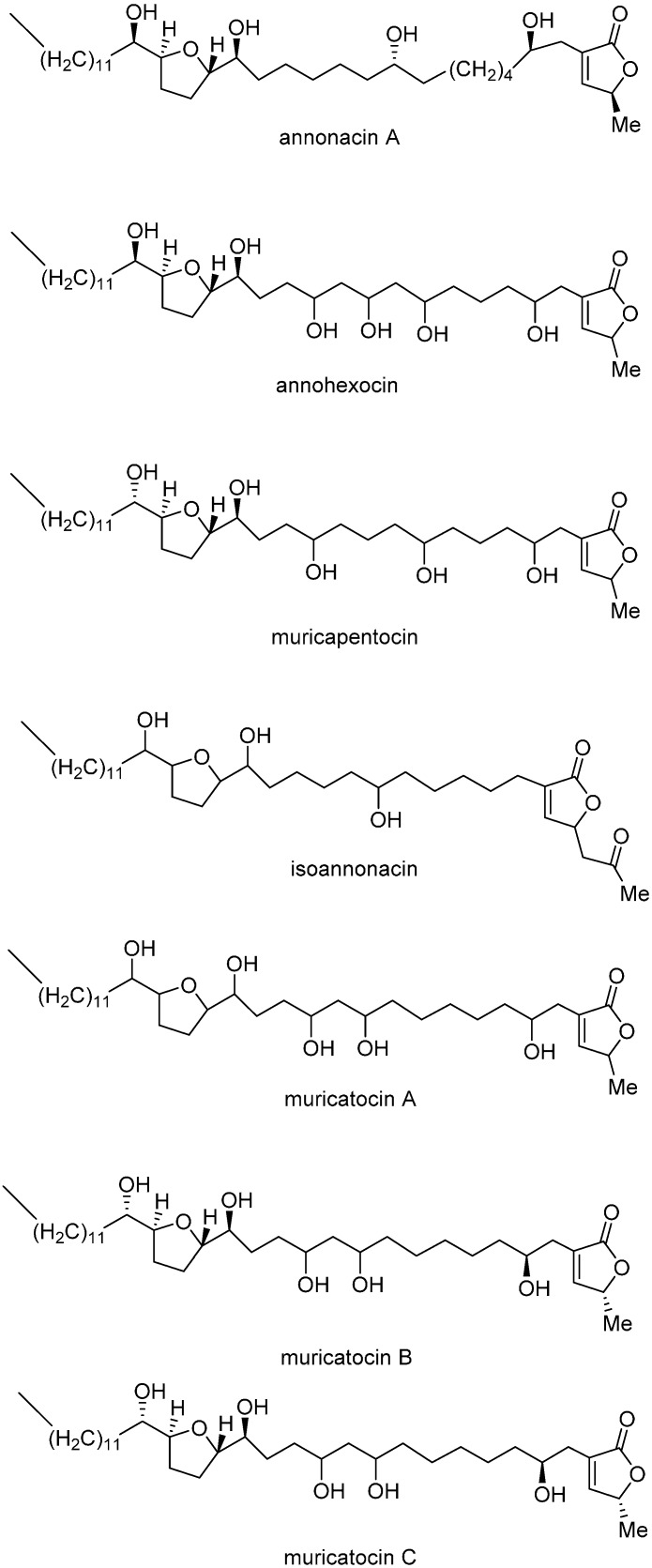

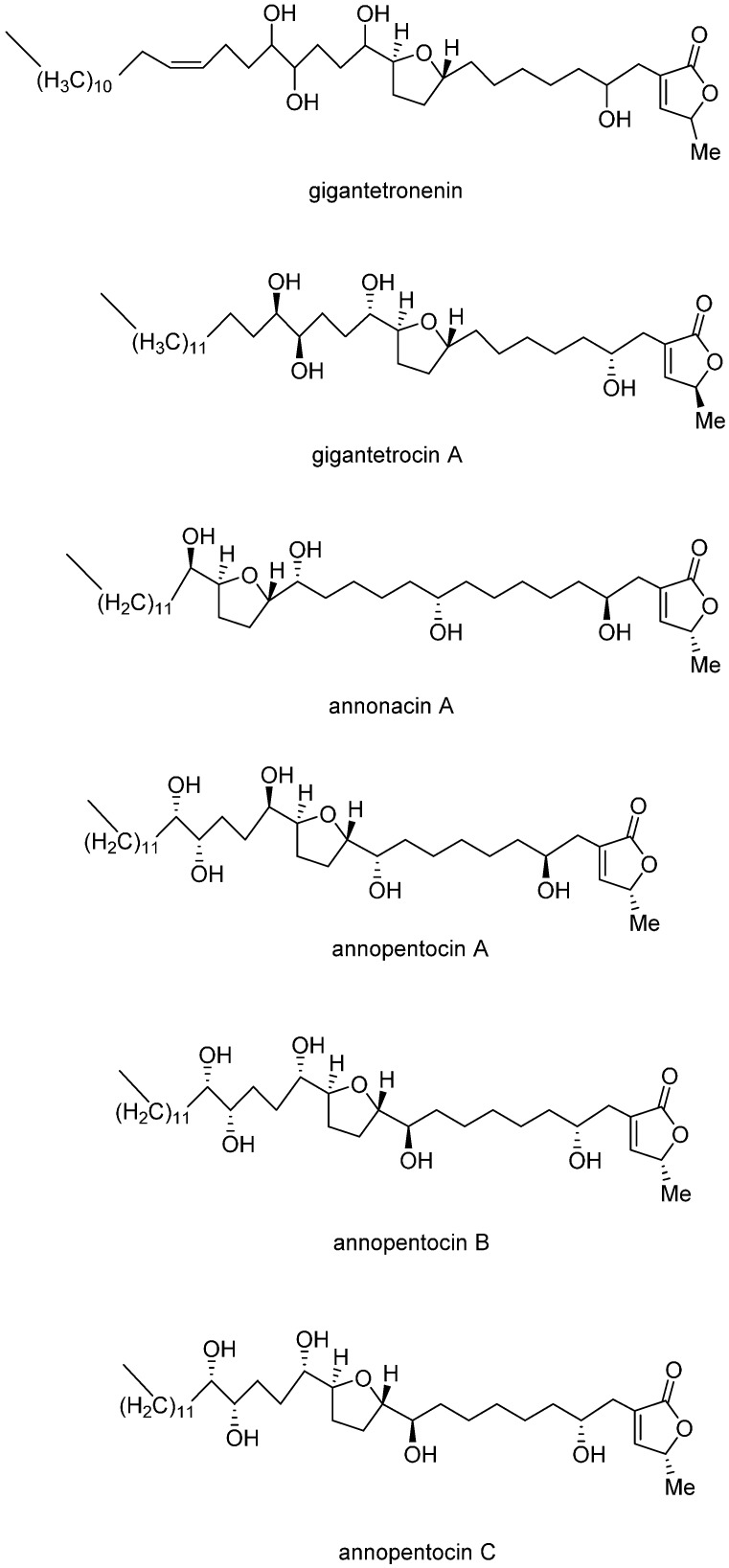

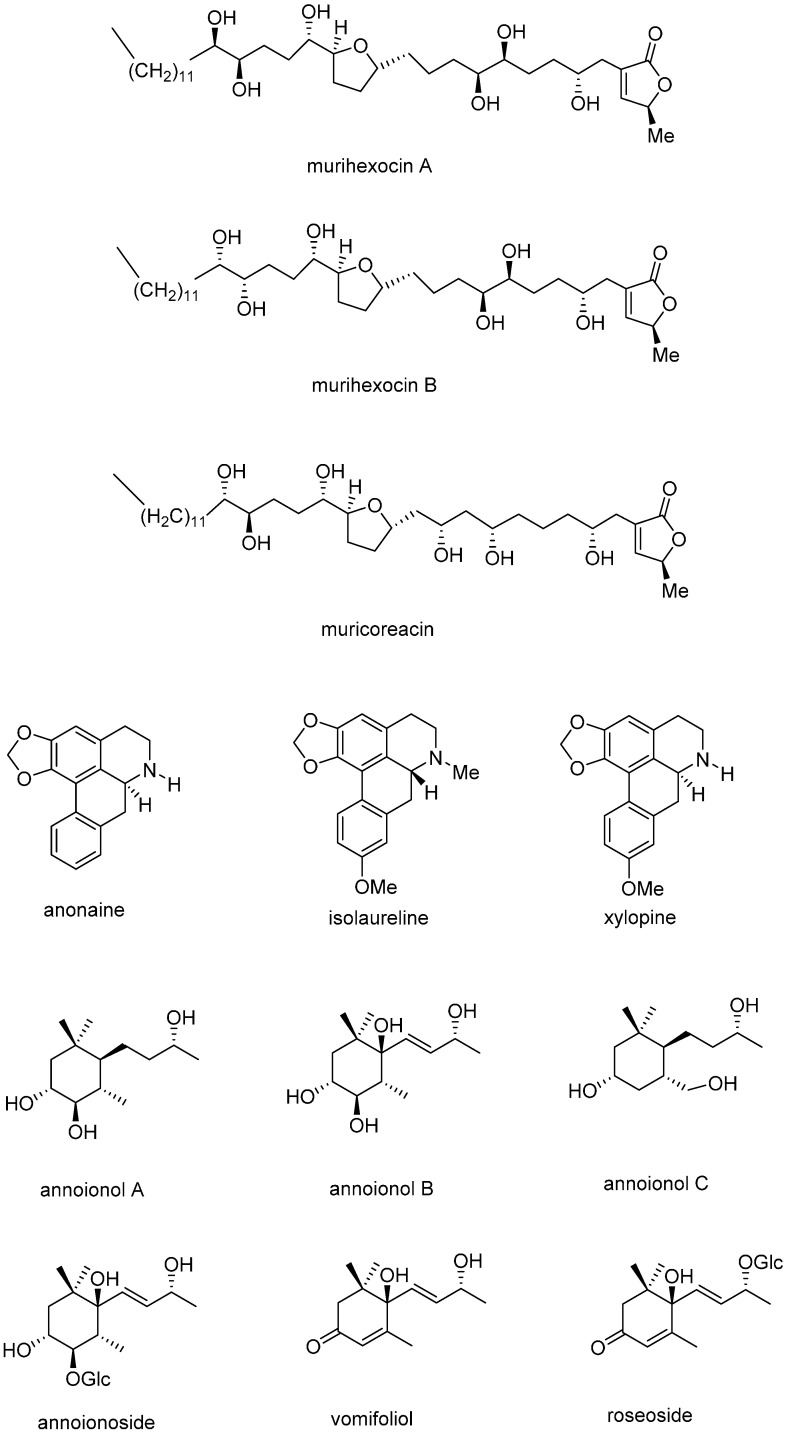

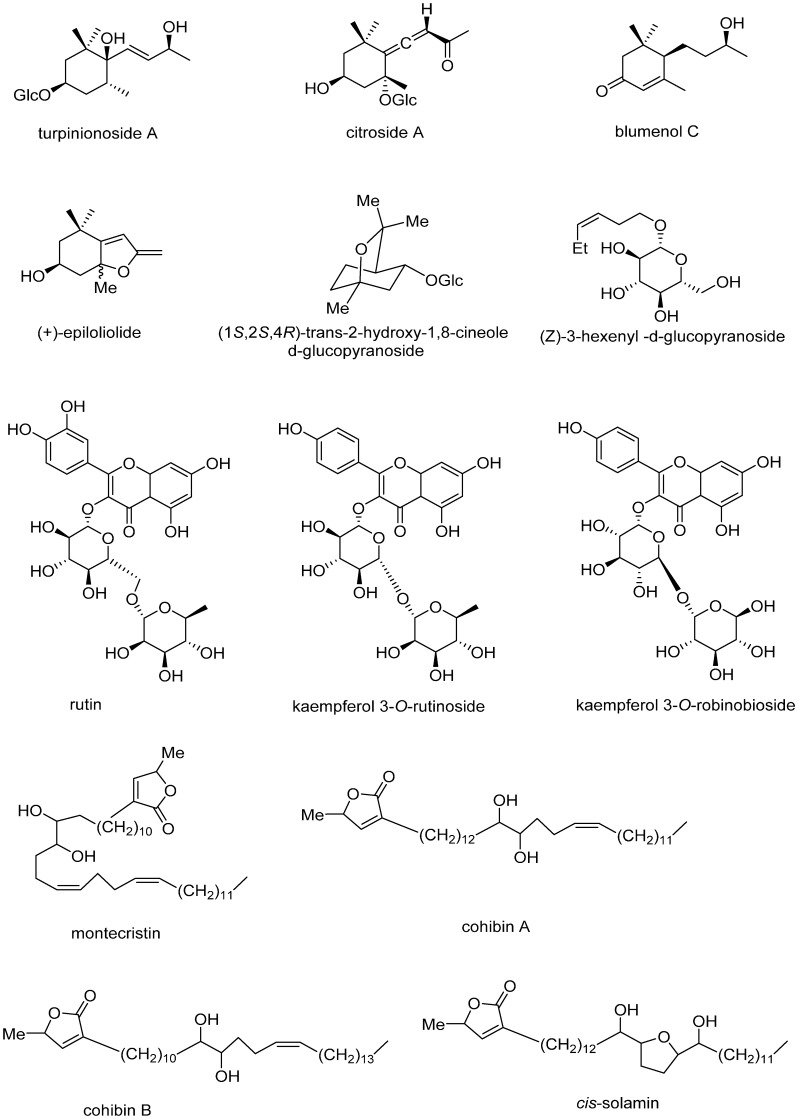

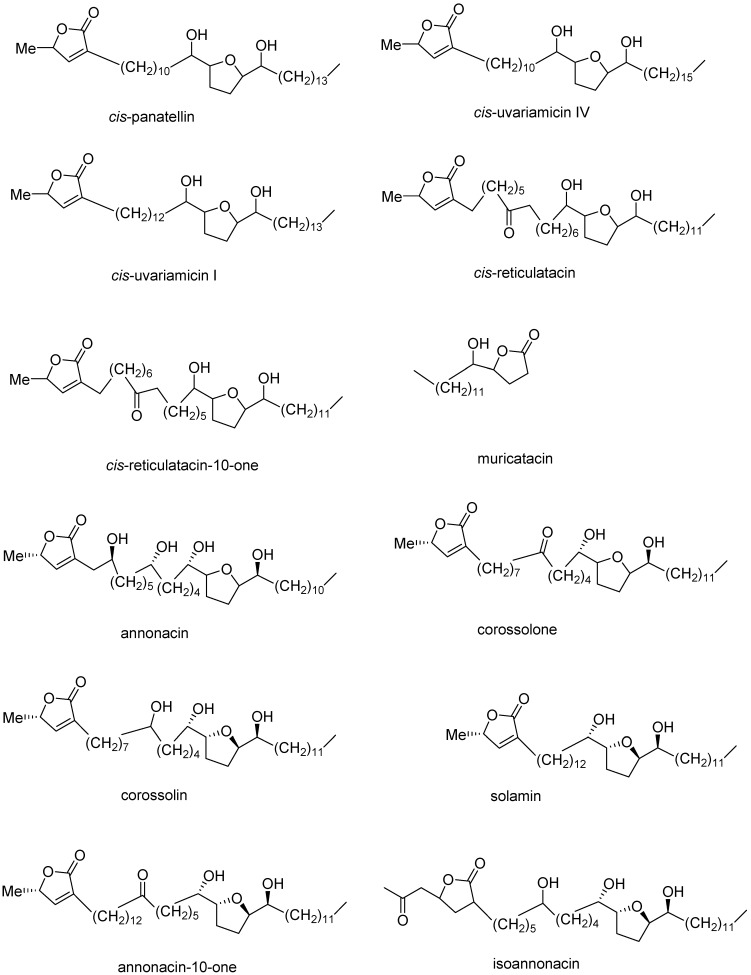

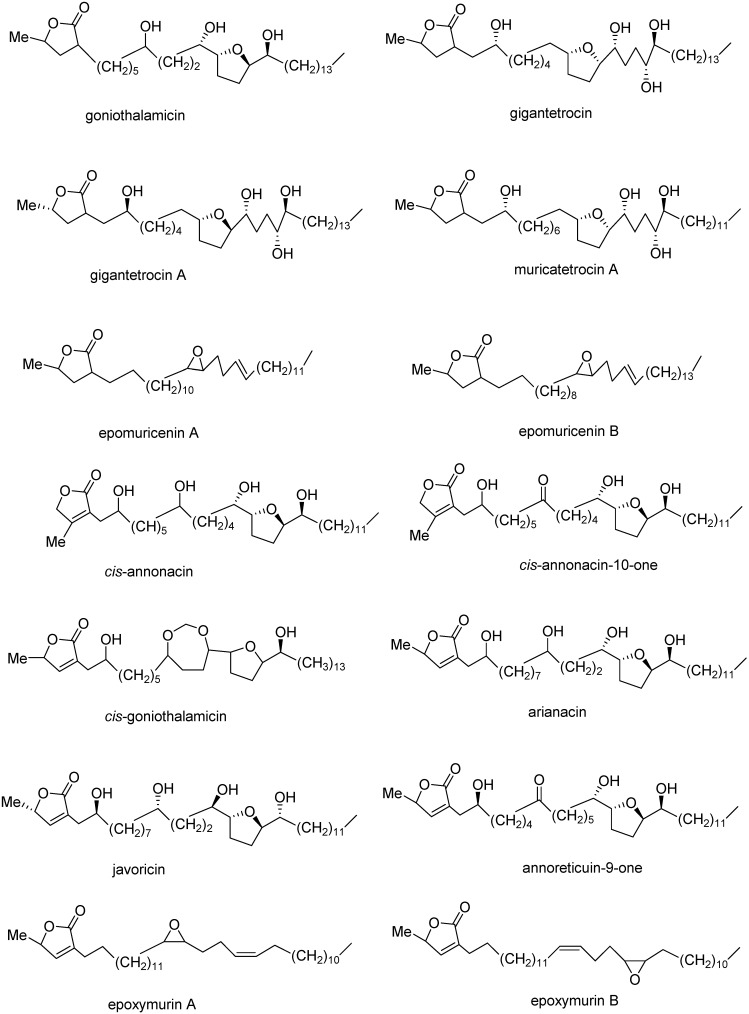
Chemical structures of the major compounds isolated from *Annona muricata*.

Extensive phytochemical evaluations on different parts of the *A. muricata* plant have shown the presence of various phytoconstituents and compounds, including alkaloids (ALKs) [4,13], megastigmanes (MGs) [14] flavonol triglycosides (FTGs) [15], phenolics (PLs) [16], cyclopeptides (CPs) and essential oils (Table 1, Figure 2) [17,18]. However, *Annona* species, including *A. muricata*, have been shown to be a generally rich source of annonaceous acetogenin compounds (AGEs) [19]. The presence of different major minerals such as K, Ca, Na, Cu, Fe and Mg suggest that regular consumption of the *A. muricata* fruit can help provide essential nutrients and elements to the human body [20].

**Table 1:** Chemical compounds isolated from *Annona muricata*. ALK: alkaloid; AGE: annonaceous acetogenin; MG: megastigmane; FTG: flavonol triglycoside; PL: phenolic; CP: cyclopeptide.

Plant Part	Compound	Class	Biological Activity	References
Fruits	annonaine	ALK	anti-depressive	[21,22]
Fruits	nornuciferine	ALK	anti-depressive	[21,22]
Fruits	asimilobine	ALK	anti-depressive	[21,22]
Fruits	epomusenin-A	AGE	-	[23]
Fruits	epomusenin-B	AGE	-	[23]
Fruits	epomurinin-A	AGE	-	[23]
Fruits	epomurinin-B	AGE	-	[23]
Fruits	*cis*-annoreticuin	AGE	-	[24]
Fruits	muricin J	AGE	toxicity against prostate PC-3 cancer cells	[25]
Fruits	muricin K	AGE	toxicity against prostate PC-3 cancer cells	[25]
Fruits	muricin L	AGE	toxicity against prostate PC-3 cancer cells	[25]
Fruits	cinnamic acid derivative	PL	-	[16]
Fruits	coumaric acid hexose	PL	-	[16]
Fruits	5-caffeoylquinic acid	PL	-	[16]
Fruits	dihydrokaempferol-hexoside	PL	-	[16]
Fruits	*p*-coumaric acid	PL	-	[16]
Fruits	caffeic acid derivative	PL	-	[16]
Fruits	dicaffeoylquinic acid	PL	-	[16]
Fruits	feruloylglycoside	PL	-	[16]
Fruits	4-feruloyl-5-caffeoylquinic acid	PL	-	[16]
Fruits	*p*-coumaric acid methyl ester	PL	-	[16]
Leaves, Pericarp	annomuricin A	AGE	toxicity against brine shrimp, lung A549, breast MCF-7 and colon HT-29 cancer cells	[12,26]
Leaves	annomuricin B	AGE	toxicity against brine shrimp, lung A549, breast MCF-7 and colon HT-29 cancer cells	[12]
Leaves	annomuricin C	AGE	toxicity against brine shrimp, lung A549, breast MCF-7 and colon HT-29 cancer cells	[27]
Leaves	annomuricin E	AGE	toxicity against pancreatic MIA PaCa-2 and colon HT-29 cancer cells	[28]
Leaves	annomutacin	AGE	toxicity against lung A549 cancer cells	[29]
Leaves	(2,4-*cis*)-10*R*-annonacin-A-one	AGE	toxicity against lung A549 cancer cells	[29]
Leaves	(2,4-*trans*)-10*R*-annonacin-A-one	AGE	toxicity against lung A549 cancer cells	[29]
Leaves	annohexocin	AGE	toxicity against brine shrimp and different cancer cells	[30]
Leaves	muricapentocin	AGE	toxicity against pancreatic MIA PaCa-2 and colon HT-29 cancer cells	[28]
Leaves	(2,4-*cis*)-isoannonacin	AGE	-	[31]
Leaves, Seeds	(2,4-*trans*)-isoannonacin	AGE	-	[31,32]
Leaves	muricatocin A	AGE	toxicity against lung A549 cancer cells	[31]
Leaves	muricatocin B	AGE	toxicity against lung A549 cancer cells	[31]
Leaves	muricatocin C	AGE	toxicity against brine shrimp, lung A549, breast MCF-7 and colon HT-29 cancer cells	[27]
Leaves, Seeds	gigantetronenin	AGE	-	[27,32]
Leaves, Seeds, Pericarp	annonacin A	AGE	-	[26,31,33]
Leaves	annopentocin A	AGE	toxicity against pancreatic MIA PaCa-2 cancer cells	[34]
Leaves	annopentocin B	AGE	toxicity against lung A549 cancer cells	[34]
Leaves	annopentocin C	AGE	toxicity against lung A549 cancer cells	[34]
Leaves	*cis*-annomuricin-D-one	AGE	toxicity against lung A549, colon HT-29 and pancreatic MIA PaCa-2 cancer cells	[34]
Leaves	*trans*-annomuricin-D-one	AGE	toxicity against lung A549, colon HT-29 and pancreatic MIA PaCa-2 cancer cells	[34]
Leaves	murihexocin A	AGE	toxicity against different cancer cells	[35]
Leaves	murihexocin B	AGE	toxicity against different cancer cells	[35]
Leaves	murihexocin C	AGE	toxicity against different cancer cells	[36]
Leaves	muricoreacin	AGE	toxicity against different cancer cells	[36]
Leaves	*cis*-corossolone	AGE	toxicity against human hepatoma cells	[37]
Leaves	annocatalin	AGE	toxicity against human hepatoma cells	[37]
Leaves	annocatacin B	AGE	toxicity against human hepatoma cells	[38]
Leaves	anonaine	ALK	neurotoxic	[39,40]
Leaves	isolaureline	ALK	-	[39]
Leaves	xylopine	ALK	-	[39]
Leaves	Quercetin 3-*O*-α-rhamnosyl-(1→6)-β-sophoroside	FTG	-	[15]
Leaves	gallic acid	FTG	-	[15]
Leaves	epicatechine	FTG	-	[15]
Leaves	quercetin 3-*O*-rutinosid	FTG	-	[15]
Leaves	quercetin 3-*O*-neohispredoside	FTG	-	[15]
Leaves	quercetin 3-*O*-robinoside	FTG	-	[15]
Leaves	catechine	FTG	-	[15]
Leaves	chlorogenic acid	FTG	-	[15]
Leaves	argentinine (1-*N*,*N*-dimethylethanyl-4,6-dimethoxy-3,8-dihydroxy-phenanthrene)	FTG	-	[15]
Leaves	kaempferol 3-*O*-rutinoside	FTG	-	[15]
Leaves	quercetin 3-*O*-glucoside	FTG	-	[15]
Leaves	quercetin	FTG	-	[15]
Leaves	kaempferol	FTG	-	[15]
Leaves	annonamine	ALK	-	[40]
Leaves	(*S*)-norcorydine	ALK	-	[40]
Leaves	(*R*)-4′-*O*-methylcoclaurine	ALK	-	[40]
Leaves	(*R*)-*O*,*O*-dimethylcoclaurine	ALK	-	[40]
Leaves	annoionol A	MG	-	[14]
Leaves	annoionol B	MG	-	[14]
Leaves	annoionol C	MG	-	[14]
Leaves	annoionoside	MG	-	[14]
Leaves	vomifoliol	MG	-	[14]
Leaves	roseoside	MG	-	[14]
Leaves	turpinionoside A	MG	-	[14]
Leaves	citroside A	MG	-	[14]
Leaves	blumenol C	MG	-	[14]
Leaves	(+)-epiloliolide	MG	-	[14]
Leaves	loliolide	MG	-	[14]
Leaves	(1*S*,2*S*,4*R*)-*trans*-2-hydroxy-1,8-cineole β-d-glucopyranoside	MG	-	[14]
Leaves	(*Z*)-3-hexenyl β-d-glucopyranoside	MG	-	[14]
Leaves	rutin	MG	-	[14]
Leaves	kaempferol 3-*O*-rutinoside	MG	-	[14]
Leaves	kaempferol 3-*O*-robinobioside	MG	-	[14]
Leaves	kaempferol 3-*O*-β-d-(2′′-*O*-β-d-glucopyranosyl,6′′-*O*-α-l-rhamnopyranosyl)glucopyranoside	MG	-	[14]
Roots	montecristin	AGE	-	[41]
Roots	cohibin A	AGE	-	[42]
Roots	cohibin B	AGE	-	[42]
Roots	*cis-*solamin	AGE	-	[43]
Roots	*cis-*panatellin	AGE	-	[43]
Roots	*cis-*uvariamicin IV	AGE	-	[43]
Roots	*cis-*uvariamicin I	AGE	-	[43]
Roots	*cis-*reticulatacin	AGE	-	[43]
Roots	*cis*-reticulatacin-10-one	AGE	-	[43]
Roots	chatenaytrienin 1	AGE	-	[44]
Roots	chatenaytrienin 2	AGE	-	[44]
Roots	chatenaytrienin 3	AGE	-	[44]
Roots	muridienin 3	AGE	-	[44]
Roots	muridienin 4	AGE	-	[44]
Roots	muricadienin	AGE	-	[44]
Roots	coronin	AGE	-	[45]
Roots, Fruits	sabadelin	AGE	-	[24,46]
Seeds	murisolin	AGE	-	[47]
Seeds	muricatacin	AGE	toxicity against lung A549, breast MCF7, colon HT-29 cancer cells	[48]
Seeds, Leaves, Pericarp	annonacin	AGE	neurotoxic, molluscicidal, inhibitor of mitochondrial complex I	[12,26,48,49,50,51]
Seeds, Leaves	corossolone	AGE	toxicity against oral KB cancer cells and brine shrimp larva, antileishmanial	[37,52,53,54]
Seeds	corossolin	AGE	toxicity against oral KB cancer cells and brine shrimp larva	[52]
Seeds, Roots, Leaves	solamin	AGE	toxicity against oral KB cancer and normal kidney VERO cells	[37,43,55]
Seeds	corepoxylone	AGE	-	[56]
Seeds, Leaves	annonacin-10-one	AGE	-	[12,57]
Seeds	isoannonacin	AGE	molluscicidal, anticancer	[49,57]
Seeds	isoannonacin-10-one	AGE	-	[57]
Seeds, Leaves	goniothalamicin	AGE	molluscicidal	[12,49,57]
Seeds	gigantetrocin	AGE	-	[57]
Seeds, Leaves	gigantetrocin A	AGE	toxicity against colon HT-29 cancer cells	[12,32,58]
Seeds	gigantetrocin B	AGE	toxicity against colon HT-29 cancer cells	[12,32,58]
Seeds, Leaves	muricatetrocin A	AGE	toxicity against colon HT-29 cancer cells	[58]
Seeds, Leaves	muricatetrocin B	AGE	toxicity against colon HT-29 cancer cells	[58]
Seeds, Leaves	epomuricenin A	AGE	-	[23,59]
Seeds, Leaves	epomuricenin B	AGE	-	[23,59]
Seeds	annomuricatin A	CP	-	[60,61]
Seeds	annocatacin A	AGE	toxicity against human hepatoma cells	[38]
Seeds	annomuricatin C	CP	-	[62]
Seeds	*cis*-annonacin	AGE	crown gall tumor inhibition, toxicity against brine shrimp, lung A549, breast MCF-7 and colon HT-29 cancer cells	[63]
Seeds	*cis*-annonacin-10-one	AGE	crown gall tumor inhibition, toxicity against brine shrimp, lung A549, breast MCF-7 and colon HT-29 cancer cells	[63]
Seeds	*cis*-goniothalamicin	AGE	crown gall tumor inhibition, toxicity against brine shrimp, lung A549, breast MCF-7 and colon HT-29 cancer cells	[63]
Seeds	arianacin	AGE	crown gall tumor inhibition, toxicity against brine shrimp, lung A549, breast MCF-7 and colon HT-29 cancer cells	[63]
Seeds	javoricin	AGE	crown gall tumor inhibition, toxicity against brine shrimp, A549, breast MCF-7 and colon HT-29 cancer cells	[63]
Seeds	murihexol	AGE	-	[33]
Seeds	donhexocin	AGE	-	[33]
Seeds	cohibin C	AGE	-	[64]
Seeds	cohibin D	AGE	-	[64]
Seeds	muricatenol	AGE	-	[32,65]
Seeds	2,4-*cis*-gigantetrocinone	AGE	-	[32]
Seeds	2,4-*trans*-gigantetrocinone	AGE	-	[32]
Seeds	2,4-*trans*-isoannonacin-10-one	AGE	-	[32]
Seeds	annomontacin	AGE	-	[32]
Seeds	longifolicin	AGE	toxicity against human hepatoma cells	[66]
Seeds	muricin A	AGE	toxicity against human hepatoma cells	[66]
Seeds	muricin B	AGE	toxicity against human hepatoma cells	[66]
Seeds	muricin C	AGE	toxicity against human hepatoma cells	[66]
Seeds	muricin D	AGE	toxicity against human hepatoma cells	[66]
Seeds	muricin E	AGE	toxicity against human hepatoma cells	[66]
Seeds	muricin F	AGE	toxicity against human hepatoma cells	[66]
Seeds	muricin G	AGE	toxicity against human hepatoma cells	[66]
Seeds	muricin H	AGE	toxicity against human hepatoma cells	[37]
Seeds	muricin I	AGE	toxicity against human hepatoma cells	[37]
Seeds	*cis*-annomontacin	AGE	toxicity against human hepatoma cells	[37]
Seeds, Leaves	annonacinone	AGE	-	[37]
Seeds	xylomaticin	AGE	-	[37]
Seeds	*N*-fatty acyl tryptamines	ALK	-	[32]
Seeds	annoreticuin-9-one	AGE	-	[24]
Stem barks	epoxymurin A	AGE	-	[67]
Stem barks	epoxymurin B	AGE	-	[67]
Leaves, Roots, Stems, Barks	reticuline	ALK	-	[68]
Leaves, Roots, Stems, Barks	coclaurine	ALK	-	[68]
Leaves, Roots, Stems, Barks	coreximine	ALK	-	[68]
Leaves, Roots, Stems, Barks	atherosperminine	ALK	-	[68]
Leaves, Roots, Stems, Barks	stepharine	ALK	-	[68]
Leaves, Roots, Stems, Barks	anomurine	ALK	-	[68]
Leaves, Roots, Stems, Barks	anomuricine	ALK	-	[68]

### 4.1. Essential Oil

GC and GC-MS analyses on the leaf oil of *A. muricata* collected from Cameroon showed the presence of mostly sesquiterpenes, with the major compound present being β-caryophyllene [69]. Another study on *A. muricata* collected from Vietnam identified significant volatile oil constituents of β-pinene (20.6%), germacrene D (18.1%), ρ-mentha-2,4(8)-diene (9.8%), α-pinene (9.4%) and β*-*elemene (9.1%) from the leaf oil [70]. The compounds of δ-cadinene, epi-α-cadinol and α-cadinol are also other major compounds reportedly found in the leaf oil extracts [18]. The fruit pulp essential oil was found to have esters of aliphatic acids with major compounds of 2-hexenoic acid methyl ester and 2-hexenoic acid ethyl ester. However, high concentrations of mono- and sesquiterpenes, including β-caryophyllene, 1,8-cineole and linalool, were also isolated from the fruit pulp [71].

### 4.2. Annonaceous Acetogenins

AGEs are a unique class of C-35/C37 secondary metabolites derived from long chain (C-32/C34) fatty acids in the polyketide pathway. They are usually characterized by a combination of fatty acids with a 2-propanol unit at C-2 that forms a methyl-substituted α,β-unsaturated γ-lactone [72]. Since the discovery of uvaricin from *Uvaria accuminata* in 1982, more than 500 AGEs have been identified from different parts of the plants in the Annonaceae family [73,74]. Due to the special structures and extensive biological activities, AGEs have attracted significant scientific interest in recent years. Various biological activities have been reported for AGEs, including antimalarial, antiparasitic and pesticidal activities [72,75]. However, the biological activities of AGEs are primarily characterized with toxicity against cancer cells and inhibitory effects against the mitochondrial complex I (mitochondrial NADH: ubiquinone oxidoreductase) [76,77]. Phytochemical investigations and biological studies on different parts of the *A. muricata* plant resulted in the identification of a wide array of AGE compounds, as summarized in Table 1. The chemical structures of the major acetogenins are shown in Figure 2. To the best of our knowledge, at the time of preparation (January 2015) of the present review over 100 AGEs have been identified in *A. muricata*.

## 5. Biological Activities

### 5.1. Anti-Arthritic Activity

*A. muricata* is among the ethnomedicines employed to treat arthritic pain. An *in vivo* study on different doses (3, 10, 30 and 100 mg/kg) of ethanolic extract from *A. muricata* leaves has investigated the anti-arthritic activity in complete Freund’s adjuvant (CFA)-induced arthritis in rats. According to the results, oral administration of the extract reduced the edema in a dose-dependent manner after two weeks of injection. Because the extract at higher doses significantly suppressed TNF-α and IL-1β expression in local tissue, the anti-arthritic activity of *A. muricata* leaves contributed to the suppression of pro-inflammatory cytokines [78]. Hence, the anti-arthritic potential of *A. muricata* was substantiated by the findings of this *in vivo* study.

### 5.2. Anticancer Activity

Plenty of studies report the significant antiproliferative effects of different extracts of the plant and isolated AGEs towards various cancer cell lines [26,79,80,81,82]; however, few of these studies have illustrated the underlying mechanism of action (Table 2). Recent *in vitro* studies were performed by our research group to determine the mechanism of action of ethyl acetate extract of *A. muricata* leaves against colon cancer cells (HT-29 and HCT-116) and lung cancer cells (A549). The leaf extract was able to induce apoptosis in colon and lung cancer cells through the mitochondrial-mediated pathway. This antiproliferative effect was associated with cell cycle arrest in the G_1_ phase [83,84]. In addition, the migration and invasion of colon cancer cells were significantly inhibited by the leaf extract. The activation of caspase 3 by the ethanolic extract of the leaves also demonstrated an apoptosis-inducing effect in myelogenous leukemic K562 cells, which was confirmed with a TUNEL assay [85].

**Table 2:** Anticancer studies on *A. muricata*.

Plant Part	Subject of Study	Effect	Reference
ethyl acetate extract of the leaves	lung A549 cancer cells	mitochondrial-mediated apoptosis, cell cycle arrest at G_1_ phase	[83]
ethyl acetate extract of the leaves	colon HT-29 and HCT-116 cancer cells	mitochondrial-mediated apoptosis, cell cycle arrest at G_1_ phase, suppression of migration and invasion	[84]
water extract of the leaves	rat’s prostate	reduction of prostate size	[86]
ethanolic extract of the leaves	breast tissues of mice	prevention of DMBA-induced DNA damage	[87]
ethanolic extract of the leaves	DMBA/croton oil induced mice skin papillomagenesis	suppression of tumor initiation and promotion	[88]
ethanolic extract of the leaves	DMH induced colon cancer	reduction of ACF formation	[89]
ethanolic extract of the leaves	K562 chronic myeloid leukemia cells	induction of apoptosis	[85]
leaves boiled in water	metastatic breast cancer	stabilization of disease	[90]
ethyl acetate of the leaves	azoxymethane induced colon cancer	reduction of ACF formation	[91]
ethyl acetate of the leaves	colon HT-29 cancer cells	bioassay-guided isolation of annomuricin E and its apoptosis inducing effect	[91]

Recent *in vitro* and *in vivo* studies were performed on the water extract of the *A. muricata* leaves against the benign prostatic hyperplasia (BPH-1) cell line and rats’ prostates. The results showed a suppressive effect on BPH-1 cells with an IC_50_ value of 1.36 mg/mL after 72 h associated with an up-regulation of Bax and a down-regulation of Bcl-2 at the mRNA level. After two months of treatment with the extract (30 and 300 mg/mL doses), the size of the rats’ prostates were decreased, which was suggested to occur through apoptosis induction [86]. This promising antitumor effect also reported in an *in vivo* study on 7,12-dimethylbenzene anthracene (DMBA)-induced cell proliferation in the breast tissues of mice. The protective effect against DNA damage induced by DMBA showed that oral administration of the *A. muricata* leaves may have protective effects towards the development of breast carcinogenesis [87]. The leaves, even at the low dose of 30 mg/kg suppressed the initiation and promotion stage of skin papillomagenesis in mice that was induced by DMBA and croton oil, respectively [88].

Moghadamtousi and colleagues [91] also examined the *in vivo* chemopreventive potential of the ethyl acetate extract of the *A. muricata* leaves against azoxymethane-induced colonic aberrant crypt foci (ACF) in rats. The oral administration of the extract at two doses (250 and 500 mg/kg) for 60 days significantly reduced ACF formation in rats, as assessed by methylene blue staining of colorectal specimens. The immunohistochemistry analysis showed that this activity was accompanied by the up-regulation of Bax and the down-regulation of Bcl-2. This significant reduction in ACF formation was also reported for the ethanolic extract of the leaves against 1,2-dimethyl hydrazine (DMH)-induced colon cancer [89]. Our study was followed by an *in vitro* bioassay-guided investigation against HT-29 cells, which led to the isolation of annomuricin E. This AGE showed mitochondrial-dependent apoptosis activity in colon cancer cells with an IC_50_ value of 1.62 ± 0.24 µg/mL after 48 h [91].

Anticancer studies on *A. muricata* were not only limited to *in vitro* and *in vivo* investigations. A case study of a 66-year old woman with a metastatic breast cancer reported that consumption of the leaves boiled in water and Xeloda resulted in stabilization of the disease [90]. These substantial anticancer and antitumor activities mentioned for *A. muricata* leaves led to tablet formulations of the ethyl acetate-soluble fraction of the leaves, which contains AGEs that can be used as a cancer adjuvant therapy [92].

### 5.3. Anticonvulsant Activity

In African countries, the decoction of the *A. muricata* leaves is traditionally used to control fever and convulsive seizures [93]. To substantiate the anticonvulsant activity of the leaves in ethnomedicine, Gouemo and colleagues [93] investigated the effect of the ethanolic extract of the leaves against pentylenetetrazol-induced tonic-clonic seizures in mice. The result showed that the plant extract at 100 and 300 mg/kg doses significantly decreased the incidence and the mortality rate of tonic seizures. Administration of the extract to mice also lengthened the onset of clonic seizures. This study showed that a subsequent bioassay-guided investigation may lead to the isolation of a bioactive compound that can be used as an anticonvulsant drug.

### 5.4. Antidiabetic and Hypolipidemic Activity

The chronic disease of diabetes mellitus afflicts a large proportion of people all around the world. Therefore, an effective natural adjuvant therapy would be blindingly beneficial to diminish diabetic complications and augment the quality of life for diabetic patients. Due to the traditional application of *A. muricata* against diabetes, several studies have investigated this potential *in vivo*. Adeyemi and colleagues [94] reported that daily intraperitoneal injection of streptozotocin-induced diabetic Wistar rats with the methanol extract of *A. muricata* leaves (100 mg/kg) for two weeks significantly reduced their blood glucose concentration from 21.64 to 4.22 mmol/L [94]. In addition, the extract at the same dose significantly decreased the serum total cholesterol, low-density lipoprotein, triglyceride and very low-density lipoprotein cholesterol [95].

Based on the ethnopharmacological application of *A. muricata* leaves against diabetes in Cameroon, another similar study examined the aqueous extract of the leaves against streptozotocin-induced diabetes in rats and reported the same promising antidiabetic activities. This activity was explained by its antioxidant and hypolipidemic potentials and protective effects against pancreatic β-cells [96]. Histopathological examination showed that the leaf extract caused the regeneration of β-cells in the pancreas islets [5,97]. The stem bark ethanolic extract also demonstrated promising antidiabetic and hypolipidemic activities against alloxan- induced diabetic rats. Treatment with the extract (150 and 300 mg/kg) to rats for 14 days lowered the increased blood glucose and was associated with a reduction in cholesterol and triglyceride levels [98].

### 5.5. Anti-Inflammatory and Anti-Nociceptive Activities

Oral treatment in rats with *A. muricata* ethanolic leaf extracts (10, 30, 100 and 300 mg/kg) significantly reduced carrageenan-induced edema in rat paws by 79% in a dose-dependent manner, exhibiting its anti-inflammatory activities [99]. This anti-inflammatory effect was accompanied by reductions in the leukocyte migration and exudate volume [7]. Oral administration in mice with the same extract showed significant suppression of abdominal contortions induced with acetic acid (0.6% *v*/*v*), exhibiting a powerful anti-nociceptive activity [99,100]. In addition, the formalin test and paw licking and hot-plate responses also corroborated the marked analgesic effect of the *A. muricata* leaves [7,99,100]. The protective effect of the *A. muricata* leaves against Complete Freund’s adjuvant (CFA)-induced arthritis in rats and xylene-induced ear edema in mice was associated with an attenuation in the TNF-α and IL-1β protein expression, demonstrating that the leaves could be used against both acute and chronic inflammation [100]. The same assays showed the anti-inflammatory and analgesic activities for the *A. muricata* fruits, which were shown to be induced through the suppression of inflammatory mediators and interactions with the opioidergic pathway, respectively [101]. These findings demonstrated the anti-nociceptive and anti-inflammatory effects of *A. muricata* and substantiated its traditional consumption as pain killer.

### 5.6. Antioxidant Activity

Immoderate generation of intracellular reactive oxygen species (ROS) is a precursor of oxidative stress which subsequently catalyzes metabolic deficiency and cellular death through biochemical and physiological lesions [102]. The identification of antioxidants from natural products has become a matter of great interest in recent studies for their noteworthy role in nullifying the destructive effects of ROS [103,104]. DRSA, FRAP and HRSA tests on aqueous and methanolic leaf extracts of *A. muricata* revealed the marked antioxidative activities of both extracts accompanied with DNA protective effects against H_2_O_2_-induced toxicity [105]. The antioxidant activity of the *A. muricata* leaves was found to be stronger than *A*. *squamosa* and *A.* *reticulata* species as shown through different *in vitro* models, such as ABTS, nitric oxide and hydroxyl radicals [106]. The seeds and leaves of the plant are reported to possess enzymatic antioxidants, including catalase and superoxide dismutase, and non-enzymatic antioxidants, including vitamin C and E [107]. Padma and colleagues showed that the ethanolic extract of the *A. muricata* stem bark caused a reduction in lipid peroxidation induced by cold immobilization stress in the brain and liver of rats, indicating the adaptogenic potential of this plant [108,109]. The stem bark extract (200 mg/kg) also showed protective effects against oxidative stress induced by carbon tetrachloride in rats and significantly increased the oxidant levels and serum enzyme activities to near normal. The DPPH test showed the antioxidant activity of the stem bark [110]. These findings strongly suggest the potential use of *A. muricata* as a natural source of antioxidants.

### 5.7. Antihypertensive Activity

To evaluate the antihypertensive properties of *A. muricata* leaves, aqueous leaf extract (9.17–48.5 mg/kg) was administered to normotensive Sprague–Dawley rats. The results demonstrated that treatments of rats with the leaf extract significantly decreased blood pressure in a dose-dependent manner without affecting heart rates. This effect was suggested to be induced through peripheral mechanisms involving the antagonism of Ca^2+^ [111].

### 5.8. Antiparasitic Activity

Protozoal infections cause debilitating diseases, such as leishmaniasis and trypanosomiasis, which have both afflicted a noteworthy proportion of the world population. The development of resistance to empirically discovered drugs represents a major hindrance to treatment of protozoal diseases. Moreover, in case of long-term usage, toxicity and several side effects have made the available treatments more unsatisfactory. As a natural agent, *A. muricata* has been subjected to various pathogenic parasites to determine its cytotoxic effects (Table 3). The ethyl acetate leaf extract of *A. muricata* was assayed against three *Leishmania* species (PH8, M2903 and PP75) and *Trypanosoma cruzi*. Promising activity was reported with IC_50_ values lower than 25 µg/mL [112]. The same promising antileishmanial effect was reported against *L. braziliensis* and *L. panamensis* species with a toxicity effect higher than Glucantime, which was used as a positive control [26]. A bioassay-guided investigation on the *A. muricata* seeds against three *Leishmania* species, namely *donovani*, *mexicana* and *major*, led to the isolation of two AGEs as the bioactive compounds. Isolated annonacinone and corossolone elicited an EC_50_ dose of 6.72–8.00 and 16.14–18.73 µg/mL against the tested species, respectively [53]. A bioassay-guided investigation on the seeds of *A. muricata* against two forms of *L. chagasi*, promastigote and amastigote, also led to the isolation of the same bioactive AGE compounds, annonacinone and corossolone [54]. In addition, the methanolic extract of *A. muricata* seeds showed significant antiparasitic activity against the infective larvae of *Molinema dessetae*, and this activity was contributed to its isolated AGEs [113]. A recent *in vitro* investigation on *A. muricata* aqueous leaf extract was performed against *Haemonchus contortus*, a gastrointestinal parasite. The result showed 89.08% and 84.91% toxicity against larvae and eggs as assessed by larval motility and egg hatch tests. The immobilization of adult worms within 6 to 8 h of exposure to different doses of the extract revealed a promising anthelmintic activity in the leaves [114].

**Table 3:** Antiparasitic studies on *A. muricata*.

Plant Part	Subject of Study	Result	Reference
ethyl acetate extract of the leaves	*Leishmania* species (PH8, M2903, PP75), *T. cruzi*	IC_50_ values lower than 25 µg/mL	[112]
ethyl acetate extract of the pericarp	*L. braziliensis*, *L. panamensis*	toxicity effect higher than Glucantime as a positive control	[26]
methanol extract of the seeds	*L. donovani*, *L.* *mexicana*, *L. major*	bioassay-guided isolation of annonacinone (EC_50_: 6.72–8.00 µg/mL) and corossolone (EC_50_: 16.14–18.73 µg/mL)	[53]
methanol-water extract of the seeds	*L. chagasi* (promastigote amastigote)	bioassay-guided isolation of annonacinone and corossolone	[54]
aqueous extract of the leaves	*H. contortus*	toxicity against larvae (89.08%) and egg (84.91%)	[114]
pentane extract of the leaves	*P. falciparum*	toxicity against chloroquine sensitive and (IC_50_: 16 µg/mL) and resistant strains (IC_50_: 8 µg/mL)	[115]

#### Antiplasmodial Activity

Malaria, one of the most debilitating diseases, afflicts a substantial population in tropical and subtropical zones [116]. The available antimalarial drugs demonstrate varying degrees of failure due to rapid spread of parasite resistance [117]. Therefore, research into new antiplasmodial agents against the pathogenic parasites is definitely warranted. The pentane leaf extract of *A. muricata* was assayed against two strains of *Plasmodium falciparum*: the Nigerian chloroquine-sensitive strain and FcM29-Cameroon (chloroquine-resistant strain); a promising antiplasmodial effect was obtained with an IC_50_ value of 16 and 8 µg/mL after 72 h, respectively [115]. The leaf extract, also at 20 µg/mL, showed a 67% inhibition against an asynchronous F32 strain of *P. falciparum* [118]. Another study on different extracts of *A. muricata* leaves and stems also confirmed the reported cytotoxic effects against the chloroquine-sensitive (F32) and -resistant (W2) *P. falciparum* [112]. These findings substantiated the traditional use of *A. muricata* as an antimalarial agent.

### 5.9. Hepatoprotective and Bilirubin-Lowering Activity

*A. muricata* is traditionally employed to treat jaundice in Ghana. A study was conducted to determine the *in vivo* bilirubin-lowering potential of the aqueous extract of *A. muricata* leaves. This study was performed on phenylhydrazine-induced jaundice in adult rats, and the levels of direct and total bilirubin were measured in rats orally treated with 50 and 400 mg/kg of the extract. The extract at both doses caused a significant reduction to hyperbilirubinemia, which was close to normal levels [119]. In addition, the hepatoprotective effect was also reported for the aqueous extract of the leaves against carbon tetrachloride and acetaminophen-induced liver damage. Pretreatment with different concentrations of the extract (50, 100, 200, and 400 mg/kg) for 7 days prior to liver damage restored liver function toward normal hemostasis, which was shown by biochemical and histological analyses [120]. Therefore, these findings substantiated the traditional use of *A. muricata* against jaundice and showed the potential hepatoprotective activity.

### 5.10. Insecticidal Activity

Botanical insecticides can have a pivotal role in different agriculture programs, especially in small farming [121]. Due to the presence of AGEs, plants from the Annonaceae family such as *A. mucosa* and *A. sylvatica* have shown to be promising biopesticides among tropical plants [72,122]. An investigation on different *Annona* species showed the growth inhibition effect of *A. muricata* seeds and contact toxicity by topical administration to *Trichoplusia ni* larvae [122]. In another study, different extracts of *A. muricata* seeds were examined against *Sitophilus zeamais*, a detrimental pest for stored grains, using ingestion and topical assays. Promising activity was obtained from the ingestion application of hexane and ethyl acetate extracts, and this activity was contributed to the presence of AGEs in the less polar fractions [123]. By dipping and surface-protectant methods, the seed extracts revealed weevil mortality of 70% and 100% against *S. zeamais* at 20% (*v*/*v*) and 0.4% (*v*/*w*) concentrations, respectively [124].

Mosquito-controlling activity of both the aqueous and oil extracts of *A. muricata* seeds against the larvae and adults of *Aedes albopictus* and *Culex* *quinquefasciatus* demonstrated promising bioactivity with lethal concentration 50 (LC_50_) values ranging from 0.5% to 1% for larvae and 1% to 5% for adults [125]. In another study, this activity for the ethanolic extract of the leaves against *C. quinquefasciatus* was also reported with an LC_50_ value of 20.87 µg/mL after 24 h [126]. In addition, the larvae of the *Aedes aegypti* mosquito, the transmitters of dengue fever, elicited high susceptibility to the ethanolic extract of the seeds with the LC_50_ of 224.27 ppm [127]. *A. muricata* seeds showed more than five times synergistic larvicidal activity when combined with *Piper nigrum* fruit ethanolic extracts (*A. muricata* 90:10 *P. nigrum*) [128]. The fractionation analysis of the extract showed that *n*-hexane is the most active fraction with an LC_50_ of 73.77 ppm. The leaf extract of *A. muricata* also showed a time-dependent toxicity against the larvae of *Anastrepha ludens* (Mexican fruit fly) with a mortality rate of 63% to 74% [129]. Leatemia *et al.* [130] investigated the growth inhibition potential of the ethanolic seed extracts of *A. muricata* isolated from different locations against polyphagous lepidopteran *Spodoptera litura*. The surprising result showed significant differences for the growth inhibition based on the isolated locations ranging from 18% to 96% compared with the control (ethanol) [130]. The ethanolic leaf extract (1.0 g/L) showed 40%, 80% and 98% mortality against *Callosobruchus maculatus* (Fabricius) after 24, 48 and 72 h post-treatment, respectively. At the same concentration, the extract significantly decreased the oviposition of *C. maculatus* and appeared to be a promising protectant against the respective insect in stored cowpea [131]. This growing body of experimental evidence supports the idea that *A. muricata* exhibits insecticidal activity against assorted types of insects.

### 5.11. Gastroprotective Activity

Gastroprotective activity of *A. muricata* leaves was examined against ethanol-induced gastric injury. The results of the oral administration of the ethyl acetate extract (200 and 400 mg/kg) showed significant antiulcer potential, which was mediated through protective effects against gastric wall mucosal damages [100]. Immunohistochemical staining demonstrated that the leaf extract decreased the Bax protein expression and elevated the Hsp70 protein expression. The effect of the extract on the gastric tissues was accompanied with augmentation in the activity of enzymatic antioxidants and suppression of lipid peroxidation, representing the preservative effect against gastric wall mucus [132]. These findings strongly suggested the gastroprotective potential of the *A. muricata* leaves.

### 5.12. Molluscicidal Activity

To establish plant-derived molluscicides for the vector control of schistosomiasis, different parts of the *Annona* species were tested against *Biomphalaria glabrata*, both in egg masses and adult forms. Santos and colleagues, in 2001, demonstrated that the leaves of *A. muricata* possess significant toxicity against adult worms with an LD_90_ value of 8.75 ppm. Additional toxicity of the *A. muricata* leaves against snail egg masses was markedly noted among different *Annona* species [133]. A bioassay-guided investigation on the cytotoxicity of the ethanolic extract of *A. muricata* leaves against the larvae of the brine shrimp *Artemia salina* and the snail *B. glabrata* showed the potent molluscicidal activity of this plant. This study led to the isolation of three bioactive compounds of annonacin, goniothalamicin and isoannonacin [49].

### 5.13. Wound Healing Activity

Moghadamtousi and colleagues [134] investigated the wound healing activity of the ethyl acetate extract of *A. muricata* leaves (5% *w*/*w* and 10% *w*/*w*) against excisional wound healing in rats. Topical administration of the extract for 15 days demonstrated significant wound healing potential assessed by macroscopic and microscopic analyses. The anti-inflammatory effects of the extract were demonstrated during the healing process as shown by the up-regulation of Hsp70, as assessed by immunohistochemical evaluation. The antioxidant defense also fortified the wound healing activity of *A. muricata* leaves. The same experiment using the alcoholic extract of the stem bark also showed a significant reduction in the wound area from the 4th day after injury onwards [135]. These studies showed that AGEs from *A. muricata* may have potential wound healing activity against excisional wounds.

## 6. Toxicology

In 1999, a study published in the Lancet Journal discussed the possible relationship between the consumption of tropical fruits and the incidence of atypical Parkinsonism in the French West Indies [136]. In addition, the etiology of a neurodegenerative disease in Guadeloupe Island revealed a close correlation between AGE consumption and the endemic of this disease [50]. Hence, AGEs are suggested to be environmental neurotoxins responsible for neurodegenerative disorders, including Guadeloupean atypical Parkinsonism. A recent study showed that the fruit of *A. muricata* with annonacin as a major AGE may be a potential risk factor for neurodegeneration due to being a major source of exposure to AGEs [137]. In rat striatal neurons, annonacin depleted the ATP supply and interrupted the transportation of mitochondria to the cell soma, which caused cellular perturbations in the protein tau and led to a number of similar characteristics as neurodegenerative diseases [50]. It is projected that if someone consumes one soursop fruit or its nectar daily, after one year, the total amount of annonacin which was ingested is sufficient to induce brain lesions in rats through intravenous infusion [138]. Hence, excessive consumption of products from Annonaceae species should be precisely considered to prevent any neurotoxic damages.

## 7. Conclusions

*A. muricata* is a coveted tropical tree, and a wealth of phytochemical investigations have been conducted for this fruit plant. In addition to being an important source for the food industry and an indigenous medicinal plant, *A. muricata* is proven to possess a wide spectrum of biological activities. Among all former studies on this plant, the most promising activities are found to be its anticancer, antiparasitic and insecticidal activity. Because the majority of the previous studies were focused on the biological activities of the plant extract, further investigations on the biochemical and physiological functions of active compounds and the detailed mechanisms underlying these activities are completely pivotal for the development of pharmaceutical and agricultural products. In addition, clinical trials concerning the rich pharmaceutical potential of *A. muricata* have been markedly neglected in previous studies. Several reports on the neurodegenerative effects of *A. muricata* and its isolated AGEs are completely perplexing, and further research is crucial to distinguish all the compounds contributing to this effect and determine the threshold of these compounds at which this effect is caused. This review is hoped to be a source of enlightenment and motivation for researchers to further perform *in vitro*, *in vivo* and clinical investigations on the biological activities of *A. muricata* to gain insight into developing new agricultural and pharmaceutical agents.
